# Clinical characteristics, treatment status and complications in women with tube ovarian abscess and endometriosis: a retrospective study

**DOI:** 10.1186/s12905-020-01119-x

**Published:** 2021-03-18

**Authors:** Hui Li, Yan Zhao, Xiao-hong Chang, Yue Wang, Hong-lan Zhu

**Affiliations:** 1 Department of Obstetrics and Gynecology, Peking University People’s Hospital, Beijing, 100044 China; 2 Gynecological Oncology Center, Peking University People’s Hospital, Beijing, 100044 China

**Keywords:** EM, Tube ovarian abscess, Infertility, Adverse complications

## Abstract

**Background:**

The aim of our present study was to investigate the clinical characteristics, treatment status and complications in women with endometriosis (EM) and tube ovarian abscess (TOA) to determine the possible association between TOA and EM.

**Methods:**

Medical records were used to analyze the clinical characteristics, treatment and complications. Twenty women who were diagnosed with TOA with EM were compared with 93 women diagnosed as having TOA without EM between January, 2008 and December, 2018.

**Results:**

In this study, TOA patients with EM were significantly more likely to have a lower age range (20–39 years) than the non-EM group [11/20 (55.0%) vs 27/93 (29.0%)]. In addition, TOA patients with EM were associated with a significantly lower rate of parity [11/20 (55.0%) vs 75/93 (80.6%)], higher rates of infertility [8/20(40%) vs 0/93(0%)] and a significantly lower incidence of elevated blood platelet counts [5/20 (25%) vs 43/93 (46.2%)]. Furthermore, women with EM had greater blood loss (347 ± 445.77 vs 204.67 ± 289.46) and an increased complication rate [3/20(15%) vs 0/93(0%)]. Among the 3 patients who had complications in the EM group, 2 patients had septic shock and 1 patient had intestinal obstruction. And 1 case who had septic shock followed by IVF treatment. There was no significance difference on other factors.

**Conclusions:**

The present study indicated that EM did not increase the difficulty and time of treatment in patients with TOA, but increased bleeding during surgery and serious complications. It is suggested that doctors should pay more attention to postoperative treatment and nursing in women with TOA and EM, especially those who have a history of recent infertility treatment and related procedures.

**Supplementary information:**

**Supplementary information** accompanies this paper at 10.1186/s12905-020-01119-x.

## Background

Tube ovarian abscess (TOA) is a complication of pelvic inflammatory disease (PID) that occasionally involves the adjacent tissues and organs in the pelvic cavity [1]. The presentations of TOA vary and include fever, lower abdominal-pelvic pain, and vaginal discharge. TOA often leads to long-term complications, such as pelvic adhesion, chronic pelvic pain, infertility and ectopic pregnancy [2]. The occurrence of TOA is affected by many factors, such as sexual activity, age, diabetes and some immune deficiency diseases. Although the incidence of TOA is still unclear, approximately one-third of patients with pelvic inflammatory disease have TOA [3].

Endometriosis (EM) is a chronic inflammatory estrogen-dependent disease and an immunologically aberrant condition that is prevalent in women of reproductive age. Impaired immune function is one of the causes of EM, which promotes the occurrence of infection [4, 5]. Moreover, the cyst wall is not conducive to the treatment of infection, and periodic hemorrhage of the focus promotes the spread of infection [6]. In addition, EM often causes 30–50% of affected women infertility. Patients with EM and infertility are usually treated with artificial assisted reproductive technology (ART), which may increase the risk of TOA and make it difficult to treat [7]. Thus, the risk of TOA in EM patients may be increased [6, 7]. One study reported the increased risk of PID in EM may prolong the hospitalization time and increase the difficulties in treatment [8]. However, the risk of TOA in EM still remains unclear.

Few studies have reported the presence of EM in women with TOA, which mainly analyzed and compared clinical characteristics and antibiotic treatment [8, 9]. However, whether TOA patients with EM increase the difficulty of treatment and the risk of possible complications during the process of treatment remains to be elucidated.

The aim of our present study was to investigate the clinical characteristics, treatment status and complications in women with EM and TOA to determine the possible association between TOA and EM. In this study, we conducted a retrospective study to analyze whether TOA with EM tends to be more serious and difficult to treatment.

## Methods

In this retrospective study, we reviewed medical records in 168 women who were hospitalized at Peking University People’s Hospital with the diagnosis of TOA between January 2008 and December 2018. The “gold standard” for the diagnosis of EM is laparoscopy, which show the presence of the disease and its extension [10]. The diagnosis of TOA is based on the imaging with sonography or confirmed by the laparoscopy [11]. The inclusion criteria of women with EM and controls: TOA was diagnosed based on direct visualization of a pelvic abscess during laparoscopy or exploratory laparotomy and ultrasonographic; EM were confirmed by histopathology and laparoscopy or laparotomy; women aged between 20 and 55 years. Women with EM confirmed by surgery were also ruled out from controls. In our study, patients with appendicular abscess, malignant disease, hydrosalpinx fluid, adenomyoma were excluded. We also excluded women who had TOA during perinatal period. Finally, a total of 113 records were reviewed. EM was evaluated according to the revised American Society for Reproductive Medicine (rASRM) classification [12].The type of EM combined with TOA mainly included 17 ovarian EM and 3 peritoneal EM. The study was approved by the Ethics Committee of the Peking University People’s Hospital. Data were collected from the patient’s information systems.

The following clinical variables were retrieved: (1) demographic factors:: age, menarche age, marital status, BMI, parity and infertility; (2) risk factors: history of current or previous PID, recent insertion or removal of an intrauterine device (IUD) (within 3 months), and recent pelvic surgery (within 6 weeks), history of in vitro fertilization (IVF); (3) clinical factors (at the time of index admission): abdominal pain, vaginal discharge, fever, red blood cell, white blood cell (WBC) and neutrophil counts, blood platelet counts (BPC), CA125, fibrinogen, C-reactive protein (CRP), procalcitonin (PCT), bacterial vaginosis (BV) status on cervicovaginal swabs, blood culture, bacterial culture when applicable; (4) management factors: duration of intravenous injection (IV)/oral antibiotics, duration of hospitalization, and management plan (medical, surgical), position of TOA, type of surgery (laparoscopy or laparotomy), complications. According to the diagnosis and treatment plan of pelvic abscess in our hospital, all patients were treated with intravenous antibiotics on admission. After 48 h of antibiotic treatment, if there was no significant improvement in symptoms, invasive surgery would be performed.

### Statistics

Statistics Package for Social Sciences (SPSS 20, IBM, USA) was used for statistical analysis in this study. The measurement data were analyzed with Student’s t test or the Mann–Whitney test for nonnormally distributed data. The count data are expressed as percentages, and chi-square tests were used for intergroup comparisons. P value < 0.05 was considered statistically significant.

## Results

During the 10-year study period, a total of 168 patients were initially diagnosed with TOA or pelvic abscess. Of these, 113 patients fulfilled the inclusion criteria. The final analysis was performed in 113 patients, which included 20 women with EM as the study group and 93 women without EM as the control group in Additional file 1: Fig. S1. Demographic data and clinical characteristics of the participants are shown in Table 1. Compared with the control group, women in the EM group were more likely to have lower pregnancy parities (45.0% vs 19.4%, p = 0.021) and to have a higher risk of infertility history (40.0% vs 0.0%, p = 0.000). However, there was no statistically significant differences with regard to age at menarche, menopause, marital status and BMI. We still found that the risk of TOA was significantly higher in patients with EM at the age of 20–39 (P = 0.036).

**Table 1:** The comparison of demographic factors between women endometriosis and non-endometriosis who were hospitalized for TOA

Characteristics	Endometriosis (n = 20)	No endometriosis (n = 93)	P
Age			0.036*
20–39	11/20 (55.0%)	27/93 (29.0%)	
40–55	9/20 (45.0%)	66/93 (71.0%)	
Menarche age	13.65 ± 1.67	14.23 ± 1.63	0.847
Menopause	1/20 (5.0%)	10/93 (10.8%)	0.686
Married	19/20 (95.0%)	91/93 (97.8%)	0.446
BMI	23.52 ± 3.19	23.58 ± 4.92	0.243
Parity			0.021*
0	9/20(45.0%)	18/93 (19.4%)	
≥ 1	11(55.0%)	75/93 (80.6%)	
Infertility	8/20 (40.0%)	0/93 (0.0%)	0.000*

Data are presented as mean ± SD, n (%)

*BMI* Body Mass Index, *TOA* tube ovarian abscess

*P < 0.05

As shown in Table 2, risk factors of increasing TOA were analyzed in the EM group and the non-EM group. The incidence for risk factors in the EM group compared to the non-EM group, such as history of gynecological surgery (40% vs 35.4%, p = 0.799), history of previous PID (5.0% vs 21.5%, p = 0.116), history of IVF (5.0% vs 0.0%, p = 0.177), recent insertion or removal of an intrauterine device (IUD) (40.0% vs 47.3%, p = 0.626), and the differences all had no statistical significance.

**Table 2:** Analysis risk factors of in TOA women with endometriosis and without endometriosis

Characteristics	Endometriosis (n = 20)	No endometriosis n = 93	P
History of gynecological surgery	8/20 (40.0%)	33/93 (35.4%)	0.799
History of pelvic inflammaton disease	1/20 (5.0%)	20/93 (21.5%)	0.116
IVF	1/20 (5.0%)	0/93 (0.0%)	0.177
Intrauterine ring	8/20 (40.0%)	44/93 (47.3%)	0.626

Data are presented as n (%)

*IVF* in vitro fertilization, *TOA* tube ovarian abscess

The clinical symptoms and clinical characteristics of patients in the EM group and non-EM group are listed in Table 3. Fever (35% vs 37.6%) and abdominal pain (60.0% vs 76.3%) were common clinical symptoms in the EM group and non-EM group. The results shown the symptoms did not differ between the two groups. We also compared the red blood cell count and hemoglobin concentration between the two groups. We found the red blood cell count and hemoglobin concentration had no significance difference. Moreover, most of the elevated inflammatory markers did not differ between the EM group and the non-EM group, such as WBC count (45.0% vs 41.9%, p = 0.808), fibrinogen concentration (45.0% vs 58.4%, p = 0.324), neutrophils (50.0% vs 51.6%, p = 1.000), CRP (87.5% vs 89.7%, p = 1.000), and PCT (40.0% vs 36.4%, p = 1.000) at admission, other than the BPC. The incidence of elevated BPC was notably increased than that in the control group (25% vs 46.2%, p = 0.026). Table 3 shows that 75/113 (66.4%) of women had a CA-125 level drawn on presentation, 11/14 (71.4%) women in the EM group had an elevated CA-125 level, 36/61 (59.0%) women in the non-EM group had been elevated. While, no significance difference was shown on CA-125 level.

**Table3:** Analysis of clinical symptoms and biochemical results between TOA women with and without endometriosis

Characteristics	Endometriosis (n = 20)	No endometriosis (n = 93)	P
Fever	7/20 (35.0%)	35/93 (37.6%)	1
Abdominal pain	12/20 (60.0%)	71/93 (76.3%)	0.164
Red blood cell count	3.77 ± 0.52	3.93 ± 0.61	0.404
Hemoglobin concentration	111.67 ± 16.53	110.74 ± 19.01	0.373
WBC increased	9/20 (45.0%)	39/93 (41.9%)	0.808
Blood platelet counts increased	5/20 (25.0%)	43/93 (46.2%)	0.026*
Fibrinogen increased	9/20 (45.0%)	52/93 (58.4%)	0.324
Neutrophils increased	10/20 (50.0%)	48/93 (51.6%)	1.000
CRP increased	7/8 (87.5%)	35/39 (89.7%)	1.000
PCT increased	2/5 (40.0%)	4/11 (36.4%)	1.000
CA125 increased	10/14 (71.4%)	36/61 (59.0%)	0.546
Bacteria culture positive	3/8 (27.3%)	23/46 (50.0%)	0.200
Blood culture positive	2/6 (33.3%)	2/13 (15.4%)	0.557
Secretion culture positive	0/3 (0.0%)	5/11 (45.5%)	0.258

Data are presented as mean ± SD, n (%)

*WBC* white blood cell count, *CRP* C-reactive protein, *PCT* procalcitonin, *TOA* tube ovarian abscess

*P < 0.05

In addition, bacteria cultures were sampled in 3/8 (27.3%) of the EM group and 23/46 (50.0%) of the non-EM group. Among the isolated organisms cultured from the aspirated abscess found in women with TOA, gram-negative bacteria were observed in 3 patients with EM and 13 patients without EM. Gram-negative bacteria were mainly E coli and bacteroides fragilis. Gram-positive bacteria were observed in 10 patients without EM. Gram-positive bacteria were mainly staphylococcus aureu and streptococcus. A total of 2/6 (33.3%) of blood cultures taken in the EM group and 2/13 (15.4%) in the non-EM group at admission were culture positive; 0/3 (0.0%) cervicovaginal isolate secretion cultures taken in the EM group and 5/11 (45.5%) in the non-EM group were positive. However, there was no significance difference in the bacteria cultures, blood cultures and secretion cultures.

Table 4 shows a comparison of treatment between the EM group and the non-EM group. The duration of hospital stay, total treatment time, and antibiotic treatment time did not differ between the groups. Moreover, the approach did not differ between the groups, with a similar percentage of women undergoing laparotomy as compared to laparoscopic procedures (p = 0.773). Compared with the non-EM group, the EM group was significantly associated with surgical complications during the perioperative period (15.0% vs 0.0%, p = 0.000). Three patients had perioperative surgical complications in the EM group, of whom 2 patients had septic shock and 1 patient had intestinal obstruction. We also found significant differences between the EM group and the non-EM group with respect to operative blood loss (347 ± 445.77 vs 204.67 ± 289.46, p = 0.014). There were no significant differences between the groups regarding antibiotic replacement times, abscess location, emergency surgery, or relapse.

**Table 4:** Analysis management factors of TOA in women with endometriosis and without endometriosis

Characteristics	Endometriosis (n = 20)	No endometriosis (n = 93)	P
Hospital stay	10.60 ± 8.56	11.76 ± 9.05	0.825
Total treatment time	14.40 ± 9.36	15.95 ± 11.29	0.351
Antibioti treatment time	8.10 ± 5.61	12.22 ± 8.15	0.140
Antibiotic replacement times			0.196
0	10/20 (50.0%)	30/93 (32.3%)	
≥ 1	10/20 (50.0%)	63/93 (67.7%)	
Abscess location			1.000
Tube ovary abscess	12/20 (60.0%)	55/93 (59.1%)	
Tube abscess	8/20 (40.0%)	38/93 (40.9%)	
Emergency surgery	2/20 (10.0%)	12/93 (13.3%)	0.773
Blood loss	347 ± 445.77	204.67 ± 289.46	0.014*
Laparotomy	5/20 (25.0%)	20/93 (22.2%)	0.773
Complication	3/20 (15.0%)	0/93 (0.0%)	0.005*
Relapse	1/20 (5.0%)	3/93 (3.1%)	0.547

Data are presented as mean ± SD, n (%)

*TOA* tube ovarian abscess

*P < 0.05

## Discussions

In our study, we compared the presenting characteristics and clinical outcomes of EM and non-EM groups in TOA. Among these women, we found that the abdominal pain, fever, a history of prior pelvic surgery and pelvic inflammation all have no obvious significant difference. While TOA patients with EM were more likely to have lower pregnancy parities and a higher risk of infertility history. Characteristics and treatment of women with TOA were similar regardless of whether they were complicated with EM. However, the EM group was significantly more likely to have operative blood loss and complications.

A previous retrospective analysis was performed to evaluate the prevalence of the coexistence of EM and pelvic inflammatory disease (PID), which report that the prevalence of PID in women with EM is sufficiently higher than the general population [13]. Shai E. ELIZUR also reported the prevalence of women hospitalised with PID or TOA combined with EM is also higher than the prevalence of EM [8]. Our study found the prevalence EM in the study population (20/113, 17.70%) is higher than the reported prevalence of EM (6%-10%) [14]. The results shown that our study is consistent with previous studies. It is likely that EM is a risk factor for the development of TOA.

We also analysed the type of EM combined with TOA which include ovary EM (17/20, 85%), peritoneal EM (3/20, 15%). Meanwhile, all EM patients were diagnosed with Stage III/IV EM according to rASRM classification. As previous study reported, a more severe EM (stage III and IV disease) seems to be found in women with diagnosis of PID and TOA [6]. There are two main theories to explain the reason, one is the bloody content of the endometrioma or in the peritoneal cavity. The other possible reason may be as the dysfunction of immune system. Whether the TOA associated with the pathogenesis of EM still remains unknown.

Several classic theories have been proposed regarding the exact origin of EM. One of the most accepted theory is Sampson’s retrograde menstruation, which occurs in more than 90% of menstruating women [15]. Accumulating evidence suggests the multifactorial nature of the pathogenesis of EM, such as immune cells, adhesion molecules, extracellular matrix metalloproteinase and pro-inflammatory cytokines activate/alter peritoneal microenvironment, which created the conditions for differentiation, adhesion, proliferation and survival of ectopic endometrial cells [16–18]. The inflammatory conditions of TOA may support the point of view of new theories about the pathogenesis of EM.

TOA is one of the most serious complications of PID and potentially life-threatening. Early diagnosis and appropriate treatment are important for preventing long-term morbidity, such as tubal factor infertility, ectopic pregnancy, chronic pelvic pain. Recent studies reported that the predictive markers of TOA [19, 20]. Lee S.W et al. reported that increased CRP and CA-125 level were the independent factors predictive of TOA [19]. Demirtas et al. found that the level of CRP and ESR was statistically significantly higher in TOA group, and associated with longer duration of hospitalization and disease severity of TOA [20]. While the predictive markers for the development of TOA in EM patients still remains unclear. In this study, we compared clinical risk factors and laboratory parameters of EM and non-EM groups in TOA. Clinical risk factors and inflammatory markers, including fibrinogen, C-reactive protein (CRP) and PCT levels, were found no significant difference in the EM group than the non-EM group. While the incidence of elevated blood platelet in EM group was notably higher than the non-EM group. It has been reported that EM is considered a hormonal disease and an inflammatory condition. Simultaneously, activated platelets play an important role in the occurrence and development of EM [21, 22]. It is suggested that the result of our study is consistent with previous studies, which indicates the elevated blood platelet may be as a predictive marker.

As we all know, CA125 is a glycoprotein in the blood, which is derived from coelomic epithelia in the female genital tract, including the fallopian tube, endometrium, ovary, and peritoneum [23]. In routine clinical practice, CA125 has been investigated extensively as a biomarker of EM. Several studies have found that CA-125 serum levels were related to the occurrence of TOA and have a prognostic role during conservative parenteral antibiotic therapy [24, 25]. Whether CA125 can be used as the predictive marker for the development of TOA in EM patients. In this study, we also reported that the CA-125 levels in TOA patients with EM and non-EM. However, the results showed no obvious difference in the CA-125 level between the EM and non-EM group. It is possible that CA-125 may be elevated in benign ovarian cysts, hyperstimulation syndrome, ectopic pregnancy and fibroids, which imply it is nonspecific marker [23, 25].

This study reported that patients with TOA and EM had more serious perioperative surgical complications than the non-EM group. Among the 3 patients who had complications in the EM group, most (2/3, 66.7%) had septic shock. Through further analysis, we found that 1 case had septic shock followed by IVF treatment. Infertility treatment and related procedures undermine the integrity of the ovarian sac, which increases the risk of TOA [26]. EM is a risk factor for the infection of pelvic organs. However, the incidence of TOA after in vitro fertilization and oocyte removal is very low in patients suspected of EM by ultrasound [27]. Several case reports described sporadic cases of abscesses as serious post-ART complications [28–30]. Consumptive coagulation dysfunction is a serious complication of septic shock. If not treated in time, it may be life-threatening. Therefore, we should pay more attention to patients with TOA and EM followed by infertility treatment and the associated procedures.

The present study was not without any limitations. As this is a retrospective study, it is inherently limited by the possibility of type II error. Moreover, the sample size of patients is limited, and this was a single center study. This result needs to be further validated in a multicenter study for more patients. In addition, to be able to use antibiotics, the treating physicians may alter the management plan and may also change the clinical process.

## Conclusions

In summary, the characteristics and treatment of TOA patients with EM are similar to those without EM. Both groups tend to be antibiotic resistant and often require surgery. However, EM more often causes serious complications and surgical bleeding in TOA patients. Doctors should pay more attention to postoperative treatment and nursing in women with TOA and EM, especially those who have a recent history of infertility treatment and associated procedures.

## Supplementary information

**Additional file 1: Figure S1.** Characteristics of 168 patients hospitalised pelvic abscess between January 2008 and December 2018 at Peking University People’s Hospital. *TOA* tube ovarian abscess.

## Data Availability

The datasets generated and/or analyzed in the study are not publicly available due to potential for individual and organizational privacy to be compromised. Reasonable requests for parts of the data will be considered by the corresponding author.

## Abbreviations

TOA: Tube ovarian abscess
PID: Pelvic inflammatory disease
EM: Endometriosis
ART: Artificial assisted reproductive technology
IUD: Intrauterine device
IVF: In vitro fertilization
WBC: White blood cell
BPC: Blood platelet counts
CRP: C-reactive protein
BV: Bacterial vaginosis
PCT: Procalcitonin
IV: Intravenous injection
