# Supplementary material for: Clinical characteristics, treatment status and complications in women with tube ovarian abscess and endometriosis: a retrospective study
Source: BMC Womens Health. 2021 Mar 18;21:109. doi: 10.1186/s12905-020-01119-x (PMC7977313; doi:10.1186/s12905-020-01119-x)
Supplement: Supplementary file 1 — Additional file 1: Figure S1. Characteristics of 168 patients hospitalised pelvic abscess between January 2008 and December 2018 at Peking University People’s Hospital. TOA tube ovarian abscess. [file 12905_2020_1119_MOESM1_ESM.docx]

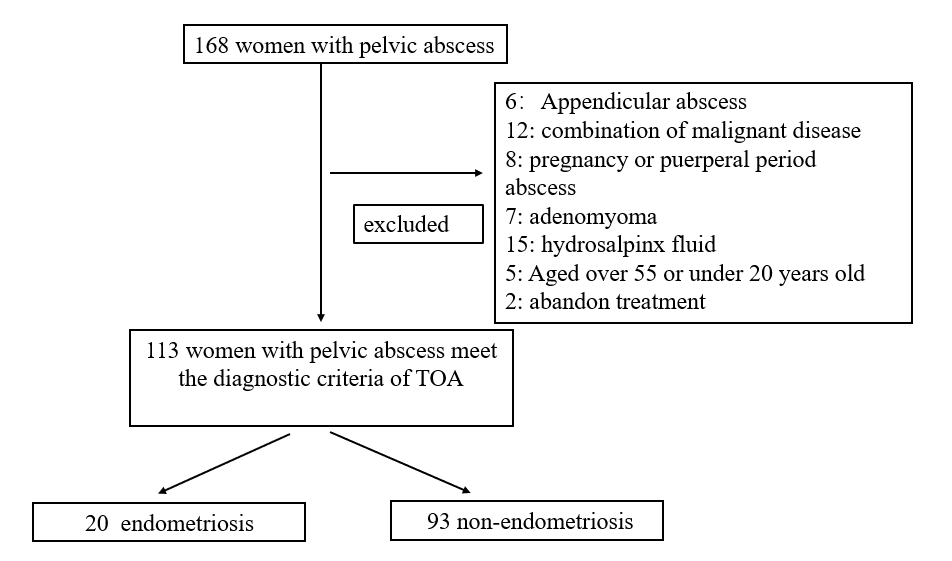


Supplement Figure1. Characteristics of 168 patients hospitalised pelvic abscess between January 2008 and December 2018 at Peking University people’ s Hospital. *TOA* tube ovarian abscess.
